# Case Report: Ultrasound “whirlpool sign” in fetal intestinal malrotation and torsion: a case-based approach to early diagnosis and intervention

**DOI:** 10.3389/fped.2025.1586328

**Published:** 2025-08-07

**Authors:** Yueyun Liu, Rongjie Zheng, Zonghua Liu, Jun Zhou

**Affiliations:** ^1^ Department of Obstetrics and Gynecology, The First Affiliated Hospital of Shandong First Medical University & Shandong Provincial Qianfoshan Hospital, Jinan, China; ^2^ Department of Obstetrics and Gynecology, Shandong First Medical University, Jinan, China; ^3^ Department of Clinical Skill Training Center, The First Affiliated Hospital of Shandong First Medical University & Shandong Provincial Qianfoshan Hospital, Jinan, China

**Keywords:** fetal ascites, intestinal malrotation, volvulus, ultrasound whirlpool sign, fetal distress, perinatal management

## Abstract

**Introduction:**

Fetal ascites is a rare condition that may indicate underlying gastrointestinal malformations, including congenital intestinal malrotation. Early recognition and timely intervention are crucial to prevent complications such as intestinal torsion and ischemic necrosis. This study reports a case of fetal intestinal malrotation presenting with isolated ascites and acute fetal distress, emphasizing the role of ultrasound and multidisciplinary management in improving neonatal outcomes.

**Case Report:**

A late-term fetus presented with reduced fetal movements. Ultrasound revealed moderate ascites, bowel dilation, and the characteristic “whirlpool sign,” suggesting intestinal malrotation with volvulus. Doppler ultrasound indicated abnormal umbilical artery blood flow, and cardiotocography (CTG) confirmed fetal distress. An emergency cesarean section was performed after a multidisciplinary consultation. Intraoperative findings revealed intestinal volvulus, and postoperative evaluation confirmed ischemic necrosis. The neonate underwent abdominal paracentesis and received comprehensive treatment, including respiratory support, antimicrobial therapy, and nutritional management. Histopathological examination confirmed localized intestinal necrosis without perforation. The infant recovered well and was discharged in stable condition.

**Conclusion:**

Fetal ascites may be an early sign of congenital intestinal malrotation and volvulus. The ultrasound “whirlpool sign” indicates intestinal malrotation and possible volvulus, aiding early detection but not confirming bowel necrosis. Prompt multidisciplinary decision-making is essential to improve perinatal outcomes and prevent severe complications.

## Graphical Abstract

Overview of the clinical process for early diagnosis and surgical intervention of fetal isolated ascites with intestinal malrotation to improve prognosis (Created by BioRender).

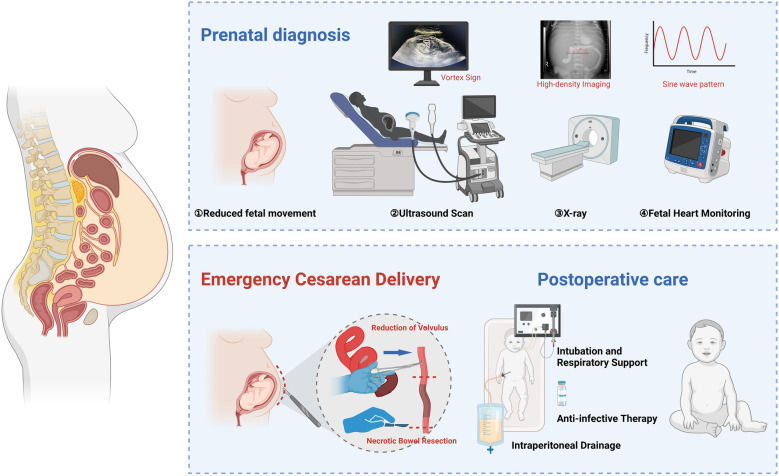

## 1 Introduction

Congenital intestinal malrotation is caused by abnormal intestinal rotation and incomplete mesenteric attachment during embryonic development (1). Although rare, it can rapidly lead to fetal intrauterine hypoxia, hemodynamic changes, and even intestinal necrosis if intestinal torsion or vascular compromise occurs, thereby increasing perinatal mortality risk (1–3). Fetal intestinal malrotation is often accompanied by ascites, with variable clinical presentations, making early diagnosis and timely intervention crucial (4). Fetal isolated ascites, a relatively uncommon ultrasound finding, may result from various causes, including chromosomal abnormalities, fetal infections, meconium peritonitis, and gastrointestinal obstruction (5–7). When ascites are accompanied by acute fetal distress, the fetus is at high risk of severe hypoxia, acidosis, and multi-organ damage, underscoring the importance of early recognition and intervention (8).

Imaging is crucial in early diagnosing fetal intestinal malrotation and its complications (9, 10). The ultrasound “whirlpool sign,” a characteristic feature of intestinal torsion, indicates abnormal mesenteric vascular twisting and serves as a key diagnostic marker for this condition (11, 12). Additionally, color Doppler ultrasound can assess hemodynamic changes in the fetal umbilical artery, ductus venosus, and middle cerebral artery, aiding in the detection of intrauterine hypoxia and serving as an important tool for predicting fetal distress (13). Cardiotocography (CTG) provides real-time evaluation of fetal heart rate baseline variability and decelerations, offering valuable insights for diagnosing fetal distress (14, 15). These imaging modalities provide essential clinical evidence for timely intervention.

The occurrence of fetal isolated ascites may indicate a severe gastrointestinal malformation, particularly intestinal malrotation and intestinal torsion (16, 17). The formation of ascites is closely associated with intestinal vascular compromise, which, if left untreated, may lead to intestinal necrosis or other life-threatening complications (18, 19). Therefore, early identification of ascites and its underlying causes, along with effective intervention, not only reduces fetal mortality but also significantly lowers the incidence of perinatal complications (20). Combining ultrasound and CTG can markedly improve the diagnostic accuracy of intestinal malrotation, optimize treatment timing, and ultimately enhance neonatal outcomes (21, 22).

This study aims to report a case of fetal isolated ascites complicated by congenital intestinal malrotation and acute fetal distress while exploring its diagnostic and therapeutic strategies. Through case analysis, we further validate the importance of the ultrasound “whirlpool sign” in early diagnosis. Additionally, we integrate color Doppler ultrasound to assess fetal hemodynamic changes and CTG for dynamic monitoring of fetal distress, proposing a multidisciplinary early intervention strategy. This study provides a novel clinical approach, particularly for high-risk fetuses with intestinal malrotation and ascites complicated by fetal distress, aiming to enhance early diagnostic accuracy and intervention efficacy, ultimately improving fetal outcomes and reducing perinatal mortality risk.

## 2 Case report

### 2.1 Case report

The patient was a 33-year-old female who presented with reduced fetal movements for half a day at 33 weeks and 3 days of gestation. She had a regular menstrual cycle (5/28 days), with her last menstrual period (LMP) on January 8, 2024. Prenatal examinations showed no abnormalities, including nuchal translucency (NT) measurement, Down syndrome screening, thyroid function tests, fetal ultrasound, and fetal echocardiography. At 24 weeks of gestation, an oral glucose tolerance test (OGTT) yielded results of 3.77–10.16–7.21 mmol/L, leading to a diagnosis of gestational diabetes mellitus (GDM), which was well controlled through dietary adjustments. The patient's obstetric history (G3P1L1A1) included a full-term vaginal delivery of a healthy female infant in 2014, with normal growth and development, and one prior early pregnancy loss due to spontaneous abortion, with no specific cause identified in the available medical records. Upon admission, vital signs were stable, and emergency laboratory tests, including infection markers, showed no abnormalities. The patient had blood type B, Rh-positive.

### 2.2 Diagnosis and preoperative management

**Figure 1:**
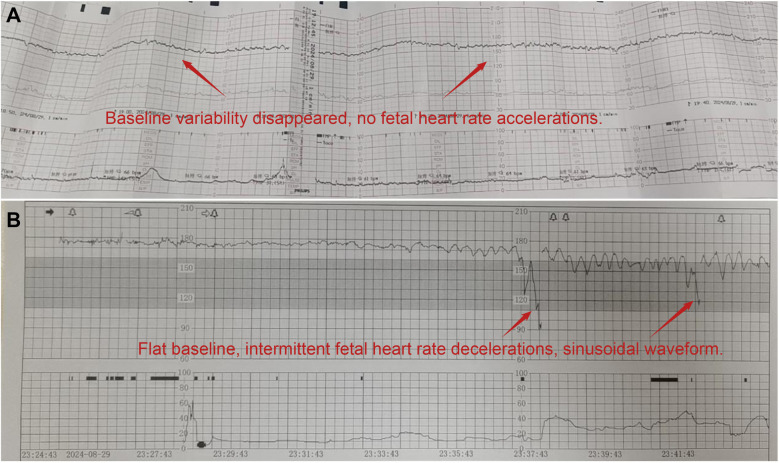
Changes and abnormalities in fetal heart rate monitoring. **(A)** Fetal heart rate monitoring (external hospital: absence of baseline variability and accelerations); **(B)** fetal heart rate monitoring (our hospital: flattened baseline with a sinusoidal pattern).

**Figure 2:**
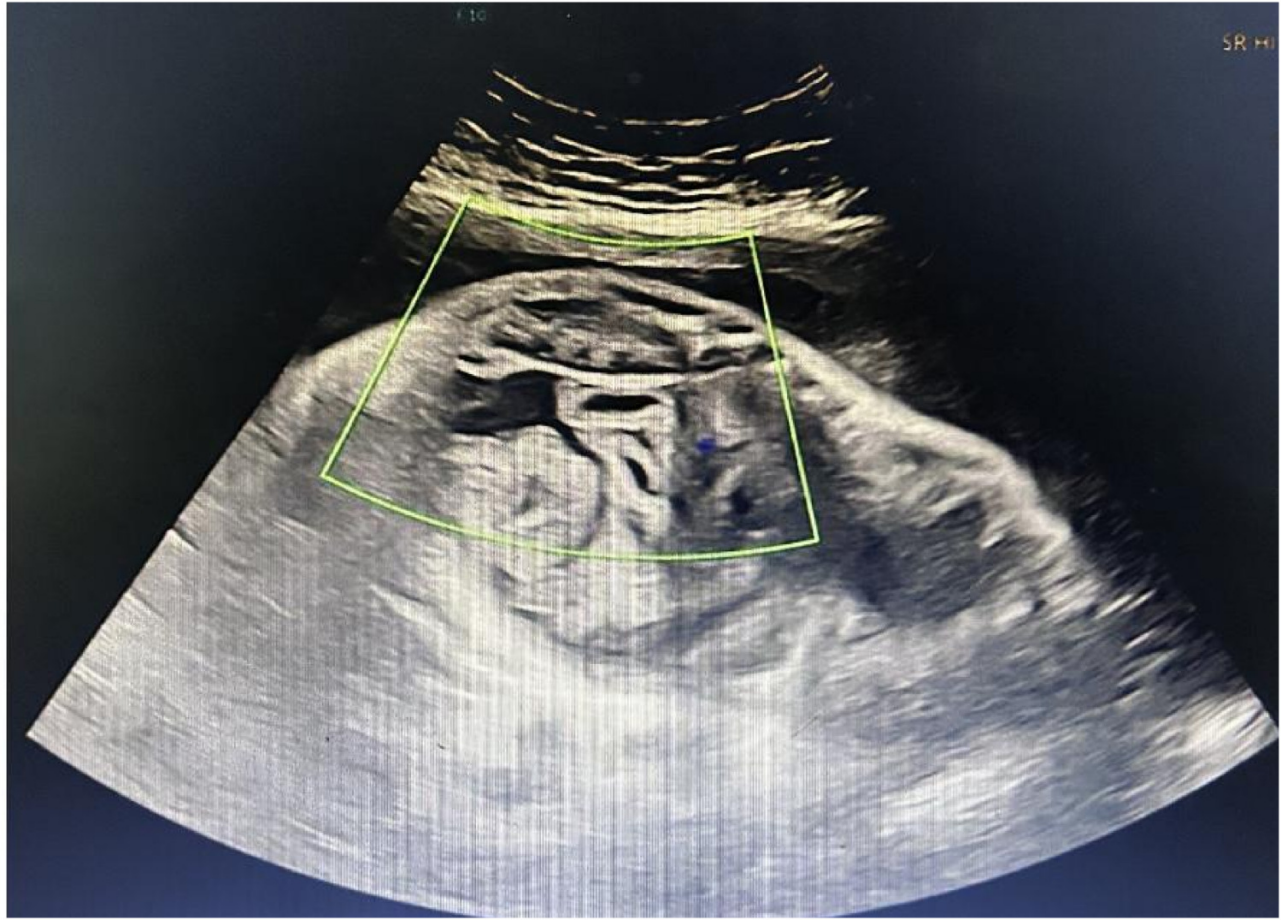
Prenatal ultrasound showing the characteristic “whirlpool sign” and fetal ascites. The image demonstrates the characteristic “whirlpool sign,” formed by the mesenteric vessels spiraling around the superior mesenteric artery, indicating fetal intestinal volvulus. A 2.3 cm anechoic area is also visible in the abdominal cavity, consistent with fetal ascites.

On August 29, 2024, the patient presented with reduced fetal movements for half a day. The specific timeline of events is detailed in Table 1 (Figures 1, 2).

**Table 1:** Timeline of preoperative management after admission.

Time	Event
2024-8-29 19:00	Fetal heart monitoring at an external hospital: absence of baseline variability, no fetal heart acceleration (Figure 1A).
2024-8-29 23:01	Patient admitted; emergency ultrasound: fetal heart rate 168–180 bpm, 2.3 cm of ascitic fluid in the abdominal cavity (Figure 2).
2024-8-29 23:24	In-hospital fetal heart monitoring: flat baseline, intermittent fetal heart deceleration, sinusoidal waveform (Figure 1B) → Emergency cesarean section performed.

The amniotic fluid was classified as grade III meconium-stained during the operation, and the umbilical cord was thin. After delivery, the newborn exhibited irregular respiration and was immediately provided with resuscitation measures, including warming, airway clearance, and positive pressure ventilation using a T-piece resuscitator (PEEP: 6 cm H_2_O, PIP: 20 cm H_2_O, FiO_2_: 21%–30%). Apgar scores were 8 at 1 min (1 point deducted for respiratory effort and muscle tone), 9 at 5 min, and 9 at 10 min (1 point deducted for respiratory effort). Given the prenatal findings, including fetal ascites and the “whirlpool sign,” a multidisciplinary team assessed the risk of intestinal volvulus. As the fetus exhibited stable vital signs and no definitive signs of intrauterine intestinal necrosis or airway compromise, an EXIT procedure was not indicated. The neonate, with a birth weight of 2.14 kg, was delivered uneventfully and immediately transferred to the neonatal unit for further evaluation and treatment.

### 2.3 Neonatal treatment process

**Figure 3:**
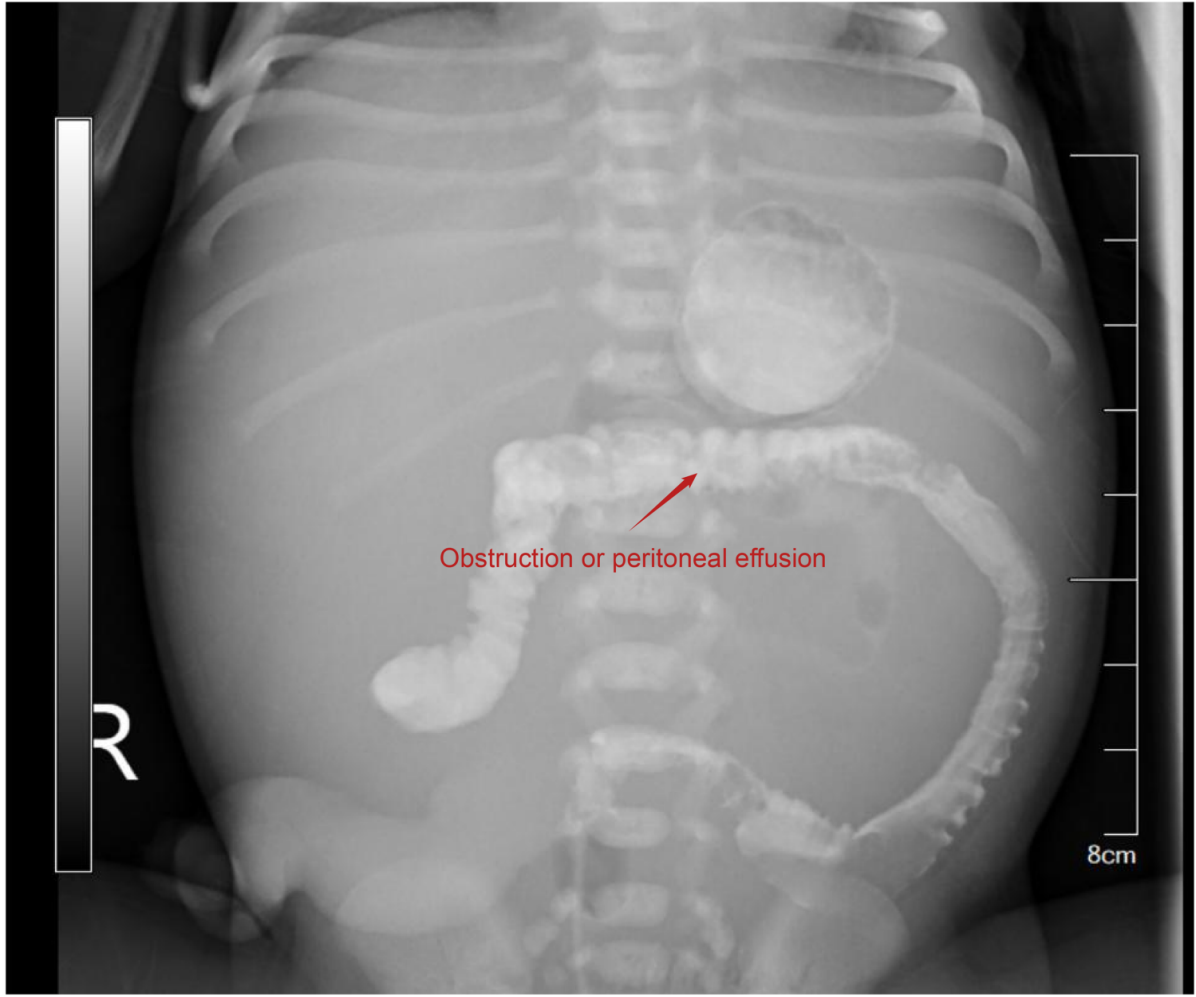
Abdominal x-ray showing high-density imaging in the gastrointestinal region. High-density imaging suggests gastrointestinal obstruction or intra-abdominal fluid accumulation.

**Figure 4:**
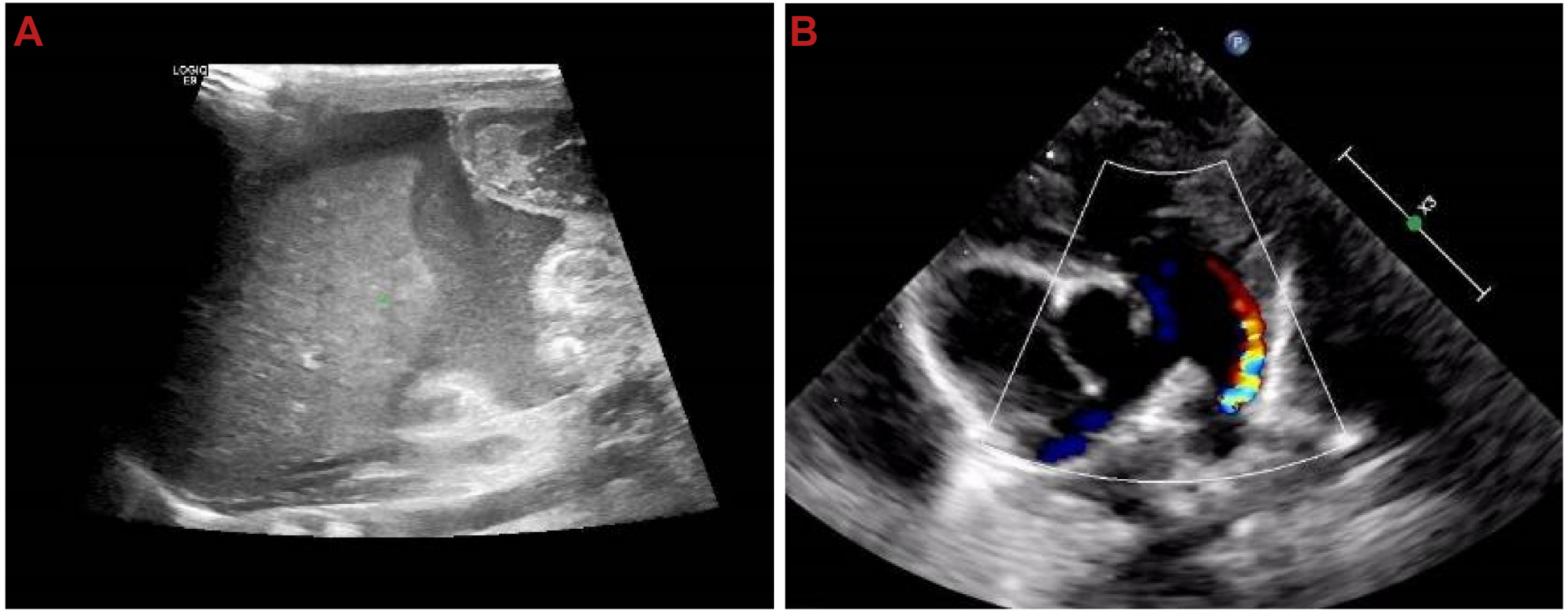
Ultrasound shows fluid-filled hypoechoic areas around the liver and spleen, as well as cardiac structural and functional abnormalities. **(A)** Hypoechoic fluid-filled areas around the liver and spleen; **(B)** neonatal echocardiography showing cardiac structural and functional abnormalities.

After admission, the newborn was identified as blood type B, Rh-positive, with a negative TORCH screening. Ascitic fluid analysis revealed turbid brown exudate, raising suspicion of peritonitis or gastrointestinal perforation. Subsequent auxiliary examinations were performed, with specific tests and results detailed in Table 2. In imaging studies, Figure 3 presents an abdominal x-ray showing high-density shadows in the gastrointestinal region, suggesting the possibility of intestinal obstruction or intra-abdominal fluid accumulation, providing a crucial clue for further diagnosis. Ultrasound examination (Figure 4A) revealed fluid-filled hypoechoic areas around the liver and spleen, further supporting the diagnosis of ascites. Figure 4B displays the results of a neonatal echocardiogram, indicating abnormal cardiac structure and function.

**Table 2:** Timeline of neonatal admission and treatment.

Time	Event
2024-8-30 Admission	Initial examination: Blood type B, Rh-positive, TORCH screening negative. Ascitic fluid analysis revealed turbid brown exudate, suggesting peritonitis or gastrointestinal perforation.
2024-8-30 10:01	Abdominal x-ray: High-density shadows in the gastric and intestinal regions, indicating obstruction or intra-abdominal fluid accumulation (Figure 3).
2024-8-30 10:46	Abdominal ultrasound: Multiple fluid-filled hypoechoic areas (liver: 38 mm, spleen: 13 mm), suggestive of peritonitis (Figure 4A).
2024-8-30 11:12	Cardiac ultrasound: Hypokinesia of the left ventricular wall, patent ductus arteriosus, patent foramen ovale, atrial septal defect, mitral regurgitation, pulmonary hypertension, and reduced left ventricular systolic function, indicating a low cardiac output state (Figure 4B).

### 2.4 Surgical procedure and management

Based on the clinical presentation of the newborn (ascites, gastrointestinal perforation, and meconium peritonitis), preoperative imaging evaluation indicated extensive small bowel dilation and compromised blood supply. To prevent disease progression and complications, an emergency resection of the necrotic bowel was performed, aiming to preserve a maximum of 30 cm of viable proximal small intestine to reduce the risk of short bowel syndrome (SBS).

**Figure 5:**
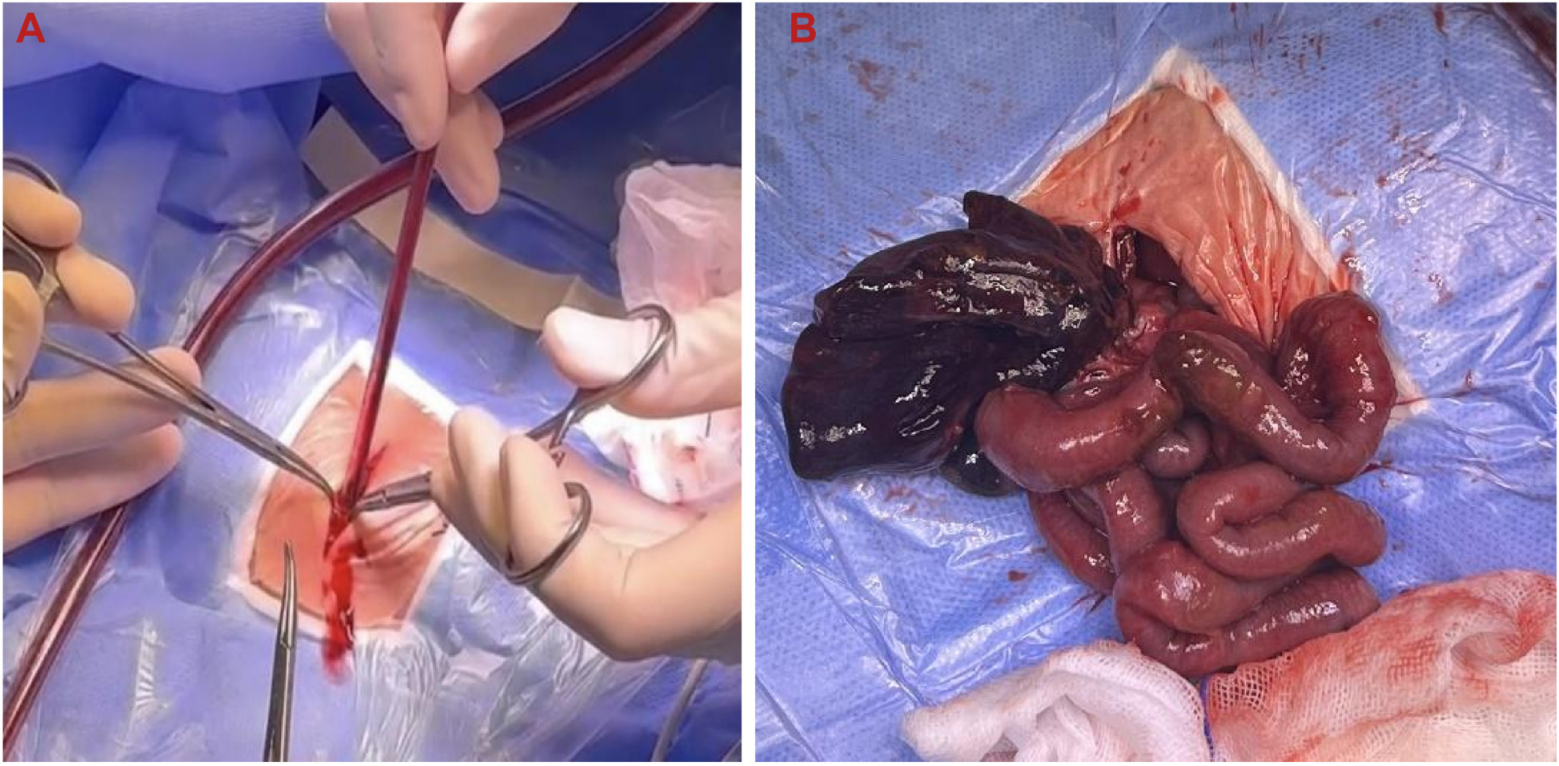
Intraoperative findings of intestinal torsion and necrosis. **(A)** Intraoperative reduction of intestinal torsion; **(B)** resected necrotic small intestine.

Intraoperative exploration revealed extensive small bowel dilation, with a 2 cm necrotic segment near the ileocecal region and a 1 cm perforation, leading to massive meconium leakage and the development of meconium peritonitis. A large amount of turbid brown exudate had accumulated in the abdominal cavity, indicating a severe inflammatory response. The mesenteric root exhibited a severe 540° torsion, causing vascular compromise and dark purple necrosis of the distal bowel. Some bowel segments were tightly adhered to the peritoneum, with visible necrotic tissue on the surface. Detailed intraoperative findings are illustrated in Figure 5A (intestinal torsion reduction) and Figure 5B (resected necrotic small bowel).

**Figure 6:**
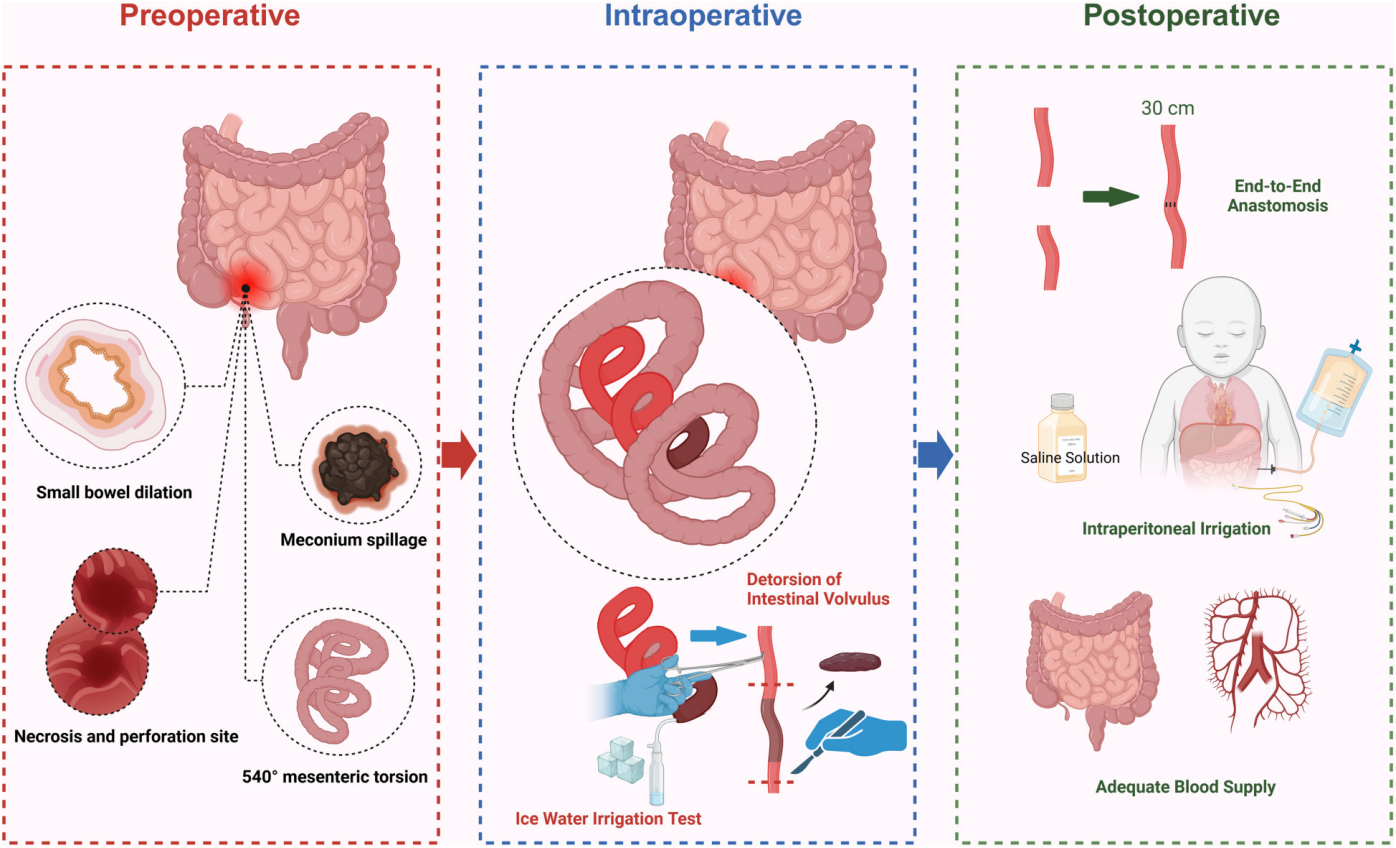
Schematic diagram of surgical procedures for intestinal necrosis resection (created by bioRender).

The surgery began with the reduction of intestinal torsion, followed by an assessment of bowel perfusion. After confirming that the necrotic bowel segment showed no signs of blood flow recovery, resection was performed, with transection at 5 cm proximal and 3 cm distal to the necrotic segment, resulting in the removal of approximately 20 cm of necrotic small intestine. An ice water immersion test was conducted to evaluate the remaining intestine's viability. Ultimately, 30 cm of the proximal small intestine was preserved. End-to-end anastomosis was performed to maintain intestinal continuity, minimizing anastomotic tension and reducing the risk of anastomotic leakage. The abdominal cavity was extensively irrigated with normal saline to remove necrotic tissue and contaminants, thereby preventing further progression of peritonitis (Figure 6).

A peritoneal drainage tube was placed postoperatively to monitor the abdominal condition and reduce the risk of infection and fluid accumulation. An individualized nutritional support plan was developed, with early initiation of total parenteral nutrition (TPN) and a gradual transition to enteral feeding. Broad-spectrum antibiotics were adjusted based on postoperative culture results to prevent secondary infections. The intraoperative diagnosis confirmed intestinal torsion, intestinal necrosis, and meconium peritonitis. The surgery was successful; the newborn remained hemodynamically stable and was subsequently transferred back to the ward for continued monitoring and treatment.

### 2.5 Postoperative follow-up results

The neonatology, gastroenterology, and nutrition departments jointly managed the newborn, with regular follow-ups at 1, 3, 6, and 12 months postoperatively and annually thereafter until 3 years of age. The follow-ups assessed growth and development, digestive function, nutritional status, imaging changes, cardiac function, and neurodevelopment.

At 1 month postoperatively, weight gain was slow, but intestinal patency was maintained. At 3 months, digestive function was normal, echocardiography showed a patent foramen ovale, and the ductus arteriosus had closed. At 6 months, body weight reached 6.8 kg, solid foods were introduced, and no signs of indigestion were observed. At 1 year, growth and development were normal, and cardiac function had fully recovered. At 2 years, the diet was normal, with no signs of intestinal obstruction or adhesions. At 3 years, body weight reached 14.2 kg, with normal language, social, and digestive function and no complications. The postoperative recovery was favorable, with no SBS or severe complications. Long-term follow-up is essential for optimizing prognosis.

### 2.6 Conclusion

The postoperative recovery was favorable, with no SBS or severe complications. Early weight gain was slow, but gradual nutritional support led to improvement. Cardiac function remained normal, with no long-term effects. This case highlights the importance of long-term postoperative follow-up, providing a reference for managing fetal intestinal torsion and intestinal necrosis. Future efforts should focus on monitoring growth and development during school age and optimizing perioperative and long-term nutritional interventions to improve prognosis.

## 3 Discussion

Fetal isolated ascites refers to fetal ascites without other ultrasound abnormalities and was first described by Winn et al. in 1990 (23). Fetal ascites can be classified into primary and secondary types. Primary fetal isolated ascites are extremely rare and are usually associated with congenital lymphatic abnormalities, leading to lymphatic obstruction, which often manifests as chylous ascites after birth. In contrast, secondary fetal isolated ascites can be divided into immune and non-immune types. Non-immune causes include fetal gastrointestinal and urinary system abnormalities, chromosomal abnormalities, and intrauterine infections. Studies have shown that 57% of non-immune ascites cases are associated with structural developmental abnormalities, 16.4% with infections, and 11.4% with genetic disorders (24, 25).

### 3.1 Diagnostic methods

The prognosis of fetal ascites depends primarily on the gestational age at diagnosis and the underlying cause. Generally, ascites diagnosed before 28 weeks are often associated with severe fetal hydrops, chromosomal abnormalities (such as T18), intrauterine infections, or genetic mutations, leading to high mortality and poor prognosis. In contrast, ascites diagnosed after 28 weeks are more likely caused by intestinal malrotation, meconium peritonitis, or transient ascites. If no chromosomal abnormalities exist, the condition may resolve spontaneously or improve with appropriate treatment, resulting in a relatively favorable prognosis. Therefore, early and accurate diagnosis combined with etiological assessment is essential for optimizing fetal outcomes.

#### 3.1.1 Prognostic assessment

The average gestational age at which fetal ascites are detected is 27.41 ± 2.98 weeks, with 55.56% diagnosed before 28 weeks and 44.44% diagnosed after 28 weeks. Factors influencing prognosis: Favorable factors: Conditions such as intestinal malrotation, meconium peritonitis, or transient ascites generally have a good prognosis if no chromosomal abnormalities are present and appropriate treatment is provided. Unfavorable factors: Conditions such as severe fetal hydrops, genetic mutation-related diseases, chromosomal abnormalities (e.g., T18), and severe intrauterine infections are associated with a poor prognosis.

#### 3.1.2 Treatment strategy

Prenatal Management: (1) Individualized assessment: The treatment approach is determined based on the underlying cause, focusing on fetal growth, amniotic fluid index, and ascites progression. (2) Intrauterine intervention: Amnioperitoneal shunt relieves pulmonary hypoplasia caused by severe ascites. (3) Ultrasound-guided fetal ascites drainage: Performed for diagnostic aspiration and to reduce ascitic pressure. Maternal corticosteroid therapy is recommended for cases at risk of fetal hydrops to promote lung maturation. (4) Consideration of pregnancy termination: If severe malformations, chromosomal abnormalities, or fetal hydrops are detected, the necessity of pregnancy termination should be comprehensively evaluated.

#### 3.1.3 Timing and mode of delivery

(1) Close monitoring of fetal heart rate and movements: If fetal distress is evident (e.g., late decelerations or sinusoidal heart rate pattern on CTG), cesarean section should be performed promptly to terminate the pregnancy. If no significant distress is observed, vaginal delivery can be considered at term or near-term, but neonatal resuscitation preparations should be made. (2) Neonatal management: A comprehensive assessment of the newborn should be conducted, including hemodynamic stability, gastrointestinal function, and ascites status. (3) Surgical intervention: Gastrointestinal abnormalities (e.g., intestinal malrotation, intestinal atresia): Exploratory laparotomy should be performed as soon as possible after birth. Meconium peritonitis: Bowel resection and repair should be performed as needed. (4) Postoperative management: Early parenteral nutrition support, with a gradual transition to enteral feeding. Strict infection monitoring, with the use of broad-spectrum antibiotics, if necessary. Respiratory support, especially in cases of pulmonary hypoplasia.

### 3.2 Literature review

We conducted a comprehensive literature search in the PubMed database using the keywords “Fetal ascites,” “Congenital malrotation of intestine,” “Intestinal torsion,” “Whirlpool sign,” and “Fetal distress.” Articles published between 2007 and 2024 were reviewed. As summarized in Supplementary Table S1, we identified nine studies most relevant to this topic and compared the treatment strategies of the present case with those reported in previous studies.

Fetal intestinal torsion is a severe prenatal gastrointestinal malformation. Early diagnosis, perinatal management, surgical treatment, and long-term follow-up directly impact neonatal survival and quality of life. This review systematically summarizes and discusses the efficacy and limitations of current treatment strategies, aiming to identify future directions for optimization.

#### 3.2.1 Importance of early diagnosis

**Figure 7:**
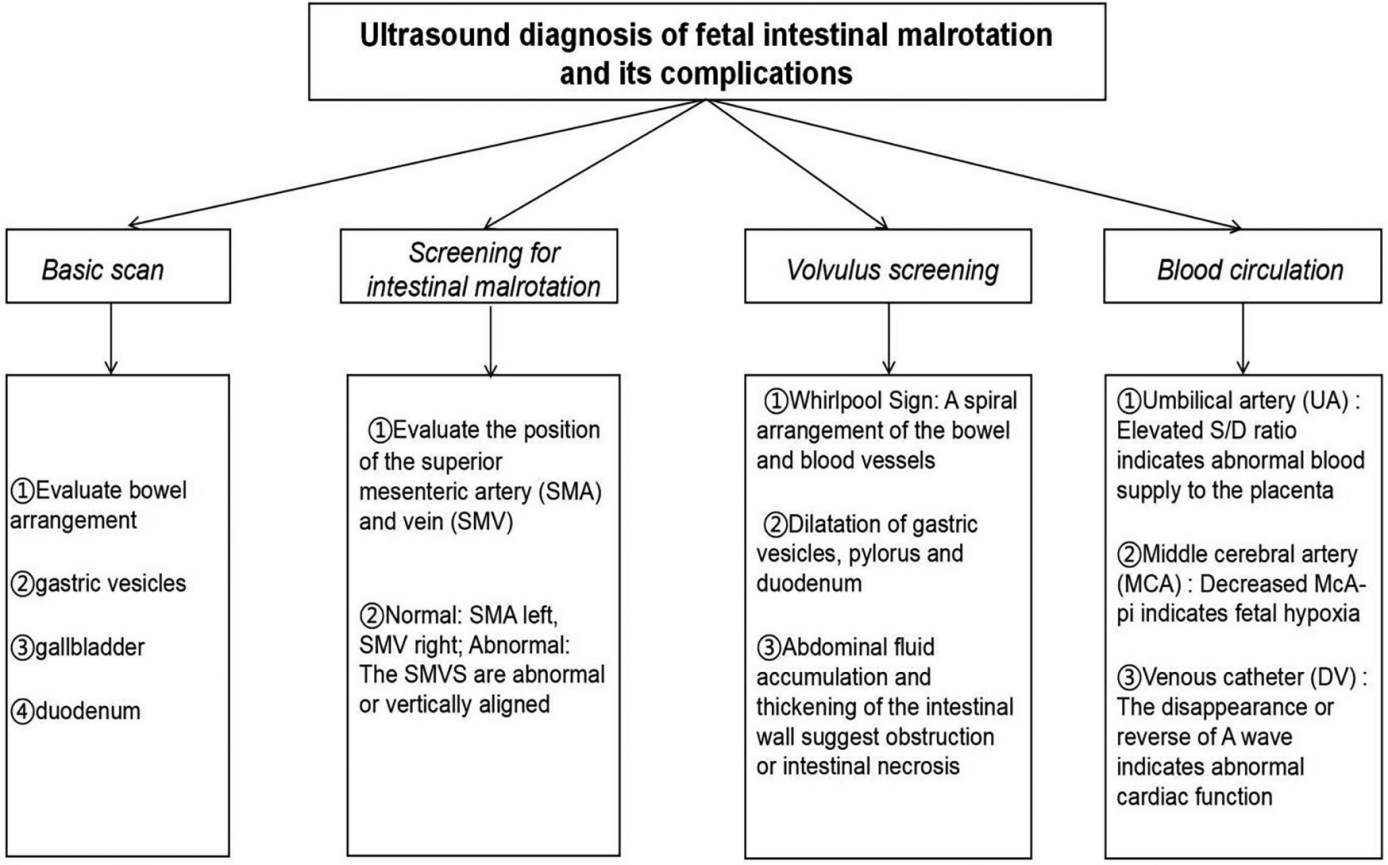
Workflow diagram for fetal ultrasound diagnosis.

Early diagnosis of fetal intestinal torsion is crucial for reducing perinatal complications and improving neonatal survival rates. The ultrasound “whirlpool sign” is a key imaging marker indicative of abnormal mesenteric vascular twisting and fetal intestinal malrotation. While it suggests a high risk of torsion, it alone cannot predict the occurrence or severity of intestinal necrosis (26). In addition, 2D/3D ultrasound (27) and fetal magnetic resonance imaging (MRI) have demonstrated high detection rates for prenatal gastrointestinal abnormalities, enabling a more accurate assessment of lesion extent and severity (28). A workflow for fetal ultrasound diagnosis is summarized in Figure 7. However, this diagnostic strategy has certain limitations. First, although the “whirlpool sign” is highly indicative, it lacks specificity, and some cases may be difficult to recognize due to intestinal dilation or fetal position. Second, image interpretation relies heavily on physician experience, leading to potential misdiagnosis or missed diagnosis due to subjective variability among radiologists (29). Additionally, although fetal MRI provides more precise anatomical imaging, its clinical application remains limited due to equipment availability, high cost, and gestational age restrictions.

#### 3.2.2 Prenatal management and perinatal decision-making

For pregnant women diagnosed with fetal intestinal torsion, perinatal management is crucial for ensuring fetal survival. Close monitoring of fetal heart rate and movements and ultrasound assessment can effectively identify intrauterine fetal distress (30). When CTG shows a sinusoidal pattern or when imaging suggests intestinal dilation and ascites, timely intervention can reduce the risk of intrauterine hypoxia and perinatal mortality (31). Studies have shown that emergency cesarean section is a key intervention for improving fetal prognosis, especially in cases of intestinal torsion complicated by fetal distress (30). However, perinatal management remains challenging. No standardized management protocol exists, and treatment approaches vary across medical institutions, leading to decision-making uncertainties. Additionally, the strong compensatory ability of the fetus may lead to an underestimation of distress, potentially delaying timely intervention. Although cesarean section improves survival rates, postoperative complications, such as intestinal injury and long-term gastrointestinal dysfunction, must be considered. Therefore, management decisions should be carefully weighed against maternal-fetal risks and long-term prognosis.

#### 3.2.3 Neonatal surgical treatment

Early surgical intervention, including bowel detorsion, resection of necrotic segments, and end-to-end anastomosis, has significantly improved neonatal survival rates (31). Timely surgery after cesarean section effectively reduces the risk of peritonitis, intestinal necrosis, and neonatal abdominal compartment syndrome. In cases of severe intestinal necrosis, preserving as much functional bowel as possible is crucial for minimizing the risk of SBS and ensuring long-term gastrointestinal function. However, surgical treatment presents several challenges. The extent of the lesion influences the success rate—if necrosis is widespread, SBS or long-term intestinal dysfunction may persist despite a successful procedure. Perioperative nutritional management and bowel function recovery remain critical concerns, as some infants may require long-term TPN, increasing the risk of hepatic injury and infections. Additionally, in extremely preterm infants or those with multiple congenital anomalies, even successful surgery may not prevent long-term impairments in growth and quality of life. Therefore, long-term postoperative follow-up is essential for optimizing prognosis.

#### 3.2.4 Prognosis and long-term management

Early diagnosis and timely intervention significantly improve neonatal survival rates while reducing the incidence of SBS and gastrointestinal complications (32). Postoperative imaging follow-up (ultrasound, x-ray, MRI) is essential for assessing intestinal obstruction, postoperative adhesions, and digestive function recovery, providing critical guidance for long-term management (27). Appropriate nutritional interventions, including breastfeeding, parenteral nutrition, and probiotic support, can promote intestinal mucosal repair and enhance absorption, contributing to postoperative recovery. However, the long-term prognosis remains challenging. Some infants may develop SBS, fat malabsorption, or refractory diarrhea, affecting growth and development. Postoperative intestinal adhesions and chronic intestinal obstruction remain major complications, with some cases requiring multiple surgical interventions, increasing medical burden and family stress. Furthermore, long-term follow-up data are currently limited, with insufficient studies exceeding 10 years. Thus, the long-term impact on digestive function and quality of life in adulthood remains unclear. Future research should focus on extended follow-up studies to optimize management strategies.

#### 3.2.5 Comprehensive evaluation of current treatment strategies

Overall, current treatment strategies have improved diagnostic accuracy and surgical success rates, but there is still significant room for optimization. Future efforts should focus on large-scale, multi-center prospective studies to establish consensus on managing fetal ascites with different etiologies and refine clinical decision-making. Additionally, imaging diagnostic criteria should be further improved to enhance the early detection rate of fetal intestinal torsion and reduce misdiagnosis and missed diagnoses. For high-risk fetuses, exploring intrauterine interventions, such as in-utero drainage and fetal surgery, may provide new therapeutic options for improving prognosis. Furthermore, postoperative nutritional support should be optimized, and management strategies for SBS should be refined to enhance long-term survival and quality of life while minimizing postoperative complications. These improvements will contribute to a more comprehensive and effective treatment system for fetal intestinal torsion.

### 3.3 Future research directions

This study provides preliminary evidence on the association between fetal isolated ascites and acute intrauterine distress, but further investigations are needed to explore etiological mechanisms, optimize diagnostic strategies, and improve treatment approaches. Future research should focus on the following areas:

Enhancing early detection: Further evaluation of the diagnostic value of fetal MRI, the ultrasound “whirlpool sign,” and Doppler flow analysis in identifying fetal intestinal torsion to improve early recognition rates. Genetic and molecular insights: Utilizing genomics and proteomics to investigate the genetic basis of congenital intestinal malrotation and related malformations, aiming to identify potential biomarkers and explore the role of genetic screening in managing high-risk pregnancies.

Multidisciplinary collaboration is critical in optimizing the perinatal management of fetal ascites, involving obstetrics, radiology, neonatology, and pediatric surgery. However, the lack of standardized diagnostic and treatment protocols leads to uncertainty in perinatal decision-making. To address this, a precise perinatal management strategy should be developed, integrating imaging techniques, biological markers, and genetic testing to establish a grading system for fetal ascites, thereby improving etiological diagnosis accuracy. Additionally, interdisciplinary coordination should be strengthened to ensure seamless integration of prenatal diagnosis, perinatal monitoring, neonatal assessment, and surgical intervention, ultimately enhancing fetal survival rates and long-term developmental outcomes. Moreover, for high-risk fetuses with conditions such as chylous ascites or meconium peritonitis, intrauterine therapeutic approaches—including in-utero drainage or pharmacological treatment—should be explored to assess their safety and efficacy, providing novel therapeutic options for severe cases of fetal ascites.

Currently, most studies on fetal ascites are single-center retrospective studies with small sample sizes, lacking high-quality, multi-center prospective research, and there is no unified consensus on management strategies. Future studies should focus on establishing large-scale prospective cohort studies through multi-center collaboration to collect cases with different etiologies, assess the impact of diagnostic and treatment strategies on prognosis, and develop personalized risk assessment models. Additionally, comparative studies should be conducted to evaluate the efficacy of expectant management *in utero* drainage, intrauterine pharmacological interventions, and the optimal timing of perinatal surgery to refine clinical decision-making and improve survival rates and long-term growth outcomes. Long-term follow-up studies should emphasize digestive, immune, and neurodevelopmental outcomes, providing evidence-based guidance for early intervention and enhancing clinical management and prognosis.

### 3.4 Conclusion

This study reports a rare case of fetal isolated ascites complicated by acute intrauterine distress, highlighting the potential association between congenital intestinal malrotation and fetal distress. The ultrasound “whirlpool sign” serves as a crucial early diagnostic marker for intestinal torsion and necrosis, providing essential guidance for clinical management. Timely intervention significantly improved fetal prognosis, emphasizing the importance of early diagnosis and rapid treatment in reducing fetal mortality. This study offers new insights into the management of similar cases.

The clinical significance of this study lies in the identification of the ultrasound “whirlpool sign” for early detection of fetal intestinal abnormalities. This enables precise intervention to reduce the risk of fetal distress and improve survival rates. This study provides practical insights for early diagnosis, individualized treatment, and perinatal decision-making, demonstrating high clinical applicability.

However, the study has certain limitations, including its single-center nature and individual case variability, which may affect the broader applicability of the findings. Future research should focus on large-scale studies to further investigate pathophysiological mechanisms, optimize diagnostic strategies, and utilize molecular biology techniques to enhance the accuracy of early screening and intervention. Additionally, multidisciplinary collaboration is crucial for improving fetal survival rates and long-term prognosis.

## Data Availability

The original contributions presented in the study are included in the article/Supplementary Material, further inquiries can be directed to the corresponding authors.

## References

[1] Yin M-D Hao L-L Li G Li Y-T Xu B-L Chen X-R. Adult-onset congenital intestinal malrotation: a case report and literature review. Medicine (Baltimore). (2024) 103:e37249. 10.1097/md.0000000000037249 38394530 PMC11309662

[2] Morris G Kennedy A Jr. Small bowel congenital anomalies. Surg Clin N Am. (2022) 102:821–35. 10.1016/j.suc.2022.07.012 36209748

[3] Wagh A Mirkhushal N Gowda T Ramteke AP G M. Twist of fate: a case series on intestinal malrotation in adult patients. Cureus. (2024) 16(10):e72763. 10.7759/cureus.72763 39618613 PMC11608110

[4] Cassart M Massez A Lingier P Absil A-S Donner C Avni F. Sonographic prenatal diagnosis of malpositioned stomach as a feature of uncomplicated intestinal malrotation. Pediatr Radiol. (2006) 36:358–60. 10.1007/s00247-005-0074-1 16465538

[5] Rădoi CL Berbecaru E-I-A Istrate-Ofițeru A-M Nagy RD Drăgușin RC Căpitănescu RG Intrauterine transmission of hepatitis C virus concomitant with isolated severe fetal ascites. Pathogens. (2022) 11:1335. 10.3390/pathogens11111335 36422587 PMC9697820

[6] Fukui K Amari S Yotani N Kosaki R Hata K Kosuga M A neonate with mucopolysaccharidosis type VII with intractable ascites. AJP Rep. (2023) 13:e25–8. 10.1055/a-2028-7784 36936745 PMC10019997

[7] Henrich W Meckies J Dudenhausen JW Vogel M Enders G. Recurrent cytomegalovirus infection during pregnancy: ultrasonographic diagnosis and fetal outcome. Ultrasound in Obstet Gyne. (2002) 19:608–11. 10.1046/j.1469-0705.2002.00705.x 12047542

[8] Sooklin L Anand AJ Rajadurai VS Chandran S. Management of large congenital chylous ascites in a preterm infant: fetal and neonatal interventions. BMJ Case Rep. (2020) 13:e235849. 10.1136/bcr-2020-235849 32878831 PMC7470640

[9] Tavasolizadeh M Dalili A. An incarcerated paraduodenal hernia of a malrotated gut in a 26-year-old man. Int J Surg Case Rep. (2024) 122:110055. 10.1016/j.ijscr.2024.110055 39043095 PMC11318467

[10] Mozaffar M Hasani M Fallah M Sobhiyeh MR Adhami F. A left paraduodenal hernia causing bowel obstruction: a case report. Gastroenterol Hepatol Bed Bench. (2013) 6:48–51. 24834245 PMC4017495

[11] Watts H Harrison R Graham-Evans K. Whirlpool sign on ultrasound imaging in a preterm infant with suspected malrotation volvulus. Arch Dis Child Fetal Neonatal Ed. (2020) 106:441. 10.1136/archdischild-2020-320804 33293277

[12] Shen AW Kothari A Flint A Kumar S. Prenatal imaging features and perinatal outcomes of foetal volvulus–A literature review. Prenat Diagn. (2022) 42:192–200. 10.1002/pd.6083 34981841

[13] Zhang Y Chi S. A rare case of small intestine torsion in pregnancy. Asian J Surg. (2023) 46:4685–6. 10.1016/j.asjsur.2023.05.082 37271647

[14] Hardalaç F Akmal H Ayturan K Acharya UR Tan R-S. A pragmatic approach to fetal monitoring via cardiotocography using feature elimination and hyperparameter optimization. Interdiscip Sci Comput Life Sci. (2024) 16:882–906. 10.1007/s12539-024-00647-6 39367993

[15] Lukhele S Mulaudzi FM Gundo R. Factors contributing to visual intrapartum cardiotocograph interpretation variation among healthcare professionals: an integrative review. PLoS One. (2025) 20:e0315761. 10.1371/journal.pone.0315761 39854512 PMC11760016

[16] Wang Q Sun K. A case report of Congenital midgut malrotation with herniation of the jejunum into a malformed omentum. Zhonghua Wei Chang Wai Ke Za Zhi. (2025) 28(1):88–9. (Chinese). 10.3760/cma.j.cn441530-20240306-00087 39971557

[17] Mandarano G Torri F Bulotta AL Bosisio M Parolini F Boroni G Acute gastric volvulus associated with wandering spleen and diaphragmatic eventration in a 5-month-old girl. J Indian Assoc Pediatr Surg. (2025) 30:81–6. 10.4103/jiaps.jiaps_122_24 39968248 PMC11832090

[18] Zivarifar H Mirbalouchzehi O Rasulizadeh M. Commentary on “cecal volvulus mimicking acute appendicitis: a rare case report” by mekoya et al. Int J Surg Case Rep. (2025) 128:111068. 10.1016/j.ijscr.2025.111068 39970610 PMC11880598

[19] Weng Y Yu C Cheng Y Zhang Y Lu S. Prenatal ultrasound diagnosis of fetal volvulus without malrotation: a case report. Eur J Obstet Gynecol Reprod Biol. (2024) 302:61–4. 10.1016/j.ejogrb.2024.08.032 39236642

[20] Yang M Zheng S Shu J Yao Z. Diagnosis of adult midgut malrotation in CT: sign of absent retromesenteric duodenum reliable. Insights Imaging. (2025) 16(1):35. 10.1186/s13244-025-01921-x 39961975 PMC11832868

[21] Schiermeier S Reinhard J Westhof G Hatzmann W. Die bedeutung der elektronischen CTG-analyse bei einem fetalen volvulus in der 32. SSW. Z Geburtshilfe Neonatol. (2008) 212:30–3. 10.1055/s-2007-990473 18293261

[22] Zeng X Li X Mo W Zhu X. Prenatal diagnosis and treatment of fetal intestinal volvulus: a case report. Medicine (Baltimore). (2025) 104:e41337. 10.1097/md.0000000000041337 39833046 PMC11749602

[23] Winn H Stiller R Grannum P Crane J Coster B Romero R. Isolated fetal ascites: prenatal diagnosis and management. Amer J Perinatol. (1990) 7:370–3. 10.1055/s-2007-999526 2222632

[24] Favre R Dreux S Dommergues M Dumez Y Luton D Oury J-F Nonimmune fetal ascites: a series of 79 cases. Am J Obstet Gynecol. (2004) 190:407–12. 10.1016/j.ajog.2003.09.016 14981382

[25] Baccega F de Lourdes Brizot M Jornada Krebs VL Vieira Francisco RP Zugaib M. Nonimmune fetal ascites: identification of ultrasound findings predictive of perinatal death. J Perinat Med. (2016) 44(2):195–200. 10.1515/jpm-2014-0326 25807579

[26] Montironi R Tosto V Quintili D Crescenzi D Battistoni GI Cobellis G Antenatal diagnosis and management of fetal intestinal volvulus: case series and literature review. JCM. (2023) 12:4790. 10.3390/jcm12144790 37510904 PMC10381374

[27] Li X Huang T Zhou M Zhang C. Prenatal diagnosis of midgut volvulus using two-dimensional and three-dimensional ultrasound. Am J Transl Res. (2022) 14:1859–67. 35422947 PMC8991126

[28] Olutoye OO 2nd Hammond JD 2nd Gilley J Beckman RM Bulathsinghala M Keswani SS Fetal malrotation with midgut volvulus: prenatal diagnosis and planning. J Pediatr Surg Case Rep. (2023) 93:102654. 10.1016/j.epsc.2023.102654 37292252 PMC10249907

[29] Ezer SS Oguzkurt P Temiz A Ince E Gezer HO Demir S Intestinal malrotation needs immediate consideration and investigation. Pediatr Int. (2016) 58:1200–4. 10.1111/ped.13075 27353636

[30] Matsushima H Katsura M Ie M Genkawa R. Intrauterine intestinal volvulus without malrotation presenting neonatal abdominal compartment syndrome. Int J Surg Case Rep. (2022) 100:107742. 10.1016/j.ijscr.2022.107742 36270210 PMC9586986

[31] Olutoye O Ohuoba E Fruhman G Zacharias N. Perinatal survival of a fetus with intestinal volvulus and intussusception: a case report and review of the literature. Am J Perinatol Rep. (2013) 03:107–12. 10.1055/s-0033-1349367 PMC3799706 24147247

[32] Su L Chen Q Zhou Y Lin W. Simultaneous cesarean section and neonatal congenital gastroschisis repair: a case report. J Sichuan Univ Med Sci. (2020) 51:732–4. 10.12182/20200960204 32975094

