# Supplementary material for: Case Report: Ultrasound “whirlpool sign” in fetal intestinal malrotation and torsion: a case-based approach to early diagnosis and intervention
Source: Front Pediatr. 2025 Aug 7;13:1586328. doi: 10.3389/fped.2025.1586328 (PMC12367481; doi:10.3389/fped.2025.1586328)
Supplement: Supplementary file 1 [file Table1.docx]

**Table S1. Literature Review of Fetal Intestinal Necrosis and Related Pathological Conditions.**

| **Journal** | **Authors** | **PMID** | **Year** | **Case Presentation** | **Diagnosis** | **Treatment** | **Outcome** |
| --- | --- | --- | --- | --- | --- | --- | --- |
| Int J Surg Case Rep | Matsushima H, Katsura M, Ie M, Genkawa R | 36270210 | 2022 | Fetal intestinal volvulus without malrotation at 36 weeks, leading to neonatal abdominal compartment syndrome. | Neonatal intestinal volvulus with extensive bowel necrosis. | Emergency cesarean section and exploratory laparotomy. | 80 cm of small intestine preserved after necrotic bowel resection. |
| AJP Rep | Ohuoba E, Fruhman G, Olutoye O, Zacharias N | 24147247 | 2013 | Fetal intestinal volvulus with type 3A jejunal atresia, detected prenatally. | Intestinal volvulus and intussusception at term. | Urgent cesarean section and exploratory laparotomy with small bowel resection and anastomosis. | Perinatal survival, infant recovered post-surgery. |
| Sichuan Da Xue Xue Bao Yi Xue Ban | Su LL, Chen QW, Zhou Y, Lin W | 32975094 | 2020 | Fetal gastroschisis detected at 35+4 weeks with absent fetal movement. | Gastroschisis confirmed via prenatal ultrasound. | Cesarean section followed by immediate neonatal surgical repair. | Good postoperative recovery, full enteral nutrition achieved. |
| J Pediatr Surg | Inoue S, Odaka A, Hashimoto D, et al. | 22008359 | 2011 | Neonatal intestinal perforation in monochorionic twins, suspected necrotizing enterocolitis. | Neonatal zygomycosis mimicking necrotizing enterocolitis. | Surgical intervention for intestinal perforation. | One twin died due to systemic infection; the other survived. |
| Case Rep Med | Klein J, Baxstrom K, Donnelly S, Feasel P, Koles P | 26612989 | 2015 | 46-year-old woman presented with acute abdominal pain, later found deceased. | Jejunal volvulus with hemorrhagic necrosis. | Postmortem diagnosis; no intervention possible. | Fatal outcome due to unrecognized volvulus. |
| J Clin Med | Montironi R, Tosto V, Quintili D, et al. | 37510904 | 2023 | Antenatal diagnosis of fetal intestinal volvulus with a literature review. | Fetal intestinal volvulus detected prenatally. | Prenatal monitoring and postnatal surgical intervention. | Improved neonatal outcomes with early diagnosis and management. |
| J Pediatr Surg Case Rep | Olutoye OO 2nd, Hammond JD 2nd, Gilley J, et al. | 37292252 | 2023 | Fetal malrotation with midgut volvulus diagnosed prenatally. | Midgut volvulus detected via prenatal imaging. | Early diagnosis facilitated postnatal surgical intervention. | Successful correction with postnatal surgical management. |
| Int J Surg Case Rep | Matsushima H, Katsura M, Ie M, Genkawa R | 36270210 | 2022 | Intrauterine intestinal volvulus without malrotation, leading to neonatal abdominal compartment syndrome. | Neonatal intestinal volvulus with extensive bowel necrosis. | Emergency cesarean section and exploratory laparotomy. | 80 cm of small intestine preserved after necrotic bowel resection. |
| Am J Transl Res | Li X, Huang T, Zhou M, Zhang C | 35422947 | 2022 | Prenatal diagnosis of midgut volvulus using 2D and 3D ultrasound. | Midgut volvulus confirmed via advanced prenatal imaging. | Postnatal surgical intervention following prenatal monitoring. | Improved neonatal outcomes with early intervention. |
